# Making gene editing a therapeutic reality

**DOI:** 10.12688/f1000research.16106.1

**Published:** 2018-12-21

**Authors:** Irina Conboy, Niren Murthy, Jessy Etienne, Zachery Robinson

**Affiliations:** 1 Bioengineering, UC Berkeley, Berkeley, CA, 94720, USA

**Keywords:** CRISPR, HDR, NHEJ, DMD, Myotonic Dystrophy, Alpha1 Antitrypsin deficiency, Clinical trials, DNA damage, AAV, nanoparticles

## Abstract

This review discusses current bottlenecks in making CRISPR-Cas9-mediated genome editing a therapeutic reality and it outlines recent strategies that aim to overcome these hurdles as well as the scope of current clinical trials that pioneer the medical translation of CRISPR-Cas9. Additionally, this review outlines the specifics of disease-modifying gene editing in recessive versus dominant genetic diseases with the focus on genetic myopathies that are exemplified by Duchenne muscular dystrophy and myotonic dystrophies.

## Successes and remaining bottlenecks in the clinical translation of CRISPR-Cas9

The treatment of genetic diseases remains one of the central challenges in medicine, where most genetic diseases—such as Duchenne muscular dystrophy (DMD), congenital heart disease, liver alpha 1 antitrypsin deficiency, familial partial lipodystrophy, and cystic fibrosis, to name a few—can be cured only if the disease-causing mutation is corrected to the wild-type sequence. Advances in genetics have revealed the molecular mechanisms that underlie these and many other genetic diseases, and CRISPR-Cas9 approaches have emerged as widely understood means to halt or even cure genetic pathologies. Additionally, CRISPR-Cas9 is being explored for treating acquired diseases, such as HIV, cancer, and hepatitis B ^1– 3^.

CRISPR-Cas9-based therapeutics have the potential to revolutionize medicine; however, developing these therapeutics requires delivering Cas9 protein and guide RNA simultaneously *in vivo*, which is challenging, since multiple components are involved. For example, all of these components are difficult to clone into a single conventional vector, thereby typically requiring multiple adenoviral-associated viruses (AAVs), to deliver one functional CRISPR-Cas9.

Importantly, even though a number of therapeutic vectors infect non-dividing cells, both the precise homology directed DNA repair (HDR) and the error-prone non-homologous end joining (NHEJ) rely on cell division: HDR is active when daughter chromatids are available (for example, in late S phase and in G _2_ phases), and NHEJ is most active in the G _1_ phase, although it can operate throughout the cell cycle ^4, 5^. For CRISPR-Cas9-mediated HDR, a donor DNA template must be provided in many cases, which further increases the number of functional components that need to be cloned into AAVs for *in vitro* and *in vivo* delivery.

CRISPR-Cas9 creates DNA double-strand breaks (DSBs) and thereby CRISPR-Cas9 therapies depend on DNA repair (ideally by HDR but in some cases NHEJ is permissive) ^6^, and since most adult tissues are composed of non-dividing differentiated and quiescent stem cells, current CRISPR-Cas9 strategies may be insufficient for correcting the mutated genes with high-enough efficiency—in enough cells to meaningfully attenuate the progression of the disease.

Additionally, regardless of the method of gene editing, *in vivo* delivery of CRISPR-Cas9 machinery remains a challenge owing to toxicity as well as immunogenicity of viral vectors: most people have or will develop antibodies against AAV, and the therapeutic CRISPR-Cas9 cargo will be fought against by the immune system and will likely be destroyed ^7– 9^. Consequentially, as of now, AAV-CRISPR-Cas9 can be introduced into patients only once in order to avoid the amplification of the adaptive immune response, thus limiting efficiency to a single dose of the treatment, which might be insufficient, particularly for large tissues such as skeletal muscle or bone. Of note, the low efficiency and low tissue selectivity of CRISPR-Cas9 hinder not only gene editing approaches but also CRISPR-Cas9a and CRISPR-Cas9i (where enzymatically inactive Cas9 is targeted to promoter/enhancer regions of specific genes by the gRNAs in order to induce the activation-a or inhibition-i of expression) ^10^. The non-viral vectors might provide a better alternative for the genome editing or the regulation of gene expression by enabling multiple administrations of CRISPR-Cas9, which is discussed in the “Future directions” section.

With respect to the gene delivery aspects of CRISPR-Cas9, continuous expression of Cas9 cDNA, which causes cumulative off-target DNA DSBs and creates insertions/deletions (indels), has inherent danger of not only gene inactivation or ectopic activation but also of oncogenic cell transformations due to the ongoing long-term genomic DNA damage. Indeed, rapid and significant genome damage, including extensive deletions and rearrangements, has recently been attributed to the effects of CRISPR-Cas9 ^11^. The danger of oncogenic transformations might be particularly high in approaches in which CRISPR-Cas9-AAV is delivered through blood circulation and is distributed to a number of tissues, including the bone marrow, gut, and liver, with their plentiful proliferating cells ^12^. Additionally, because DNA DSBs activate the guardian of the genome p53, a typical response to CRISPR-Cas9 activity is cell death by apoptosis ^13^. Approaches to inactivate p53 might improve the efficiency of CRISPR-Cas9 by preventing apoptosis ^14^, but these might also attenuate p53-dependent DNA repair, thereby increasing the probability of genomic instability.

**Figure 1.**
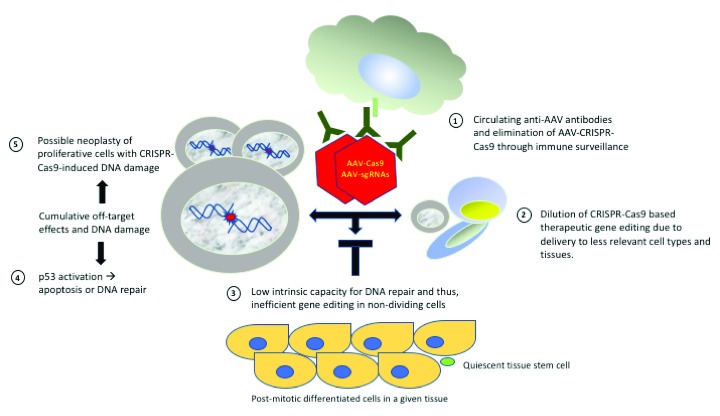
Roadblocks for clinical translation of CRISPR-Cas9. (1) Circulating anti-AAV antibodies will cause an immune response and eliminate AAV nanoparticles carrying Cas9 gRNA. (2) Owing to the imprecision of current delivery systems, there is dilution of the therapeutic CRISPR-Cas9 by less relevant cells and tissues in the body. (3) Many cells in the body, including quiescent stem cells and post-mitotic differentiated cells, have poor DNA repair, thus making CRISPR-Cas9 therapies insufficient for correcting mutated genes with high-enough efficiency. (4) CRISPR-Cas9 causes unintended off-target DNA damage which not only can lead to gene inactivation or ectopic activation but also activates p53, thus promoting cell apoptosis, particularly when DNA repair is intrinsically inefficient. (5) Proliferative cells with CRISPR-Cas9-induced damage might undergo oncogenic transformations. Despite these obstacles, CRISPR-Cas9 is moving forward in clinical trials. Several ways to overcome the bottlenecks in clinical translation of CRISPR-Cas9 are being pursued and are described in this perspective. AAV, adenoviral-associated virus; CRISPR, clustered regularly interspaced short palindromic repeats.

In the past, many promising approaches—such as gene therapy ( *in vivo* gene delivery or RNA interference [RNAi] *in vivo* gene attenuation) or cell transplantation for *in vivo* treatment of tissue degeneration—have not been widely realized to clinic, not because of a theoretical fault but because of the lack of technologies that enable their medical feasibility. It is important to fully consider the roadblocks to the clinical translation of CRISPR-Cas9 ( Figure 1) in order to overcome them and make CRISPR-Cas9 a therapeutic reality.

Even with all of the abovementioned hurdles, the disease-modifying capacity of already-developed CIRSPR-Cas9 approaches cannot be underestimated, and many research teams and biotech firms (Caribou Biosciences, CRISPR Therapeutics, Vertex Pharmaceuticals, Genedit, Editas Medicine, Intellia Therapeutics, and so on) are justly focusing on clinical trials.

There are a number of applications where patients’ bone marrow cells are removed, are CRISPR-Cas9-edited *in vitro*, are expanded in culture, and are introduced back into the patient in order to provide novel treatments for blood diseases, such as sickle cell anemia and beta thalassemia, or to engineer immune cells to resist HIV or to overcome the immune evasion of cancers ^15– 18^. It is worth mentioning that the idea to proceed with CRISPR-Cas9-based clinical trials is not without controversy ^19^; recently, the sickle cell anemia trial was put on hold by the US Food and Drug Administration (FDA) ^20, 21^.

In all of these “derive and re-transplant” approaches, the low efficiency, reliance on cell division, and tissue targeting are not a problem because proliferative hematopoietic cells are expanded and selected in culture, and even though other methods of molecular biology would achieve similar results, CRISPR-Cas9 makes genetic engineering easier. Similarly, it is much easier to create gene knockouts in cells and in experimental animals using CRISPR-Cas9, which is moving biomedicine forward at a new speed ^22^.

## Significance of CRISPR-Cas9 approaches for different classes of genetic myopathies: dominant versus recessive

In recent years, CRISPR-Cas9 approaches have been repeatedly reported in a mouse model of DMD: DMD-MDX mice ^23– 25^. DMD is an X-linked genetic myopathy that is caused by a variety of inactivating dystrophin gene mutations. The search for a disease-modifying cure for DMD is very important: it afflicts children and young adults, is currently incurable, and has devastating consequences (progressive paralysis and morbidity in the early 20s). In the reported DMD-MDX studies, the *in vivo* efficiency of dystrophin gene editing by CRISPR-Cas9 (as defined by deep sequencing of treated tissues) is about 1% to 2% with indels at about 1% of the CRISPR-Cas9 targeted sequence levels. In other words, in a mouse cell where CRISPR-Cas9 was 100% effective, 1% of indels occurred; typically, CRISPR-Cas9 is effective in about 1% to 2% of the targeted tissue in experimental MDX animals ^23– 25^. At the same time, there is an impression that CRISPR-Cas9 is massively effective because of the images that depict robust dystrophin protein in the muscles of MDX mice after CRISPR-Cas9 administrations, which are aimed at restoring the dystrophin reading frame through exon skipping ^23– 25^.

It is important to note that MDX mice that model DMD and have been widely used to test CRISPR-Cas9 editing of dystrophin have much milder disease as compared with people: MDX mice live until they are old adults and do not significantly lose their mobility. One possible reason is that the physiological workload in small mice is not comparable to that in humans; hence, the “wear-and-tear” rate and the rate of replenishment/renewal of tissue by differentiating stem (satellite) cells differ between mice and humans. Additionally, there is high telomerase activity in mice as compared with the very low activity of this enzyme and much shorter telomere lengths in people. Indeed, an experimental reduction of the telomerase activity in MDX mice resulted in more severe disease that was more similar to human DMD ^26^. Of note, the robust dystrophin protein re-expression was observed in newborn mice with their plentiful proliferative muscle precursors or young MDX mice that were injected with cardiotoxin ^23^, causing myofiber death and proliferation of myogenic cells, which are capable of effective CRISPR-Cas9. At the same time, there is a possibility that, in patients with DMD, these proliferative cells have been altered or exhausted (or both) by the disease and are not available to perform CRISPR-Cas9 ^27^.

**Figure 2.**
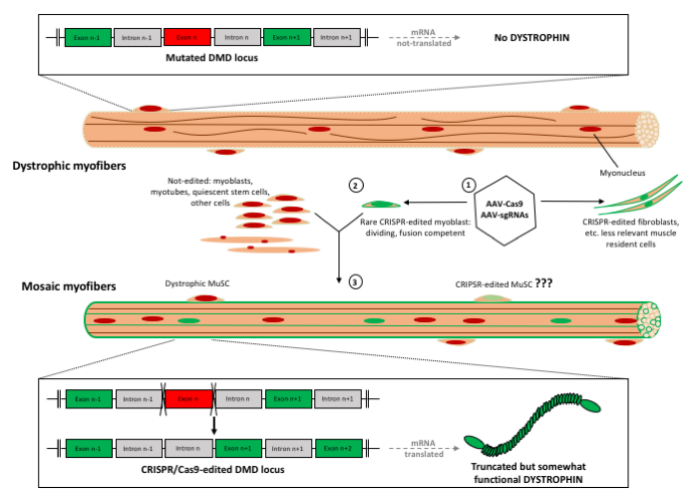
Specifics of CRISPR-Cas9 NHEJ approach to DMD. Dystrophin-negative myofibers have inactivating dystrophin gene mutation(s), resulting in the absence of dystrophin protein. Delivery of CRISPR-Cas9 (AAV-Cas9 and AAV-gRNAs) (1) leads to editing of the mutated exon (that is, removing a premature stop codon, red box) in rare muscle precursor cells, myoblasts, which can divide, migrate, and fuse with a number of myofibers (2). Many non-dividing cells in the muscle, including the quiescent MuSCs (muscle stem cells) and myofibers, are not efficiently edited by CRISPR-Cas9, and other muscle-resident cells, such as fibroblasts, might be edited. Truncated but partially functional dystrophin protein is delivered to clusters of myofibers (green outlines) through fusion events with the rare CRISPR-Cas9-edited myoblasts, where 1,000s copies of dystrophin mRNAs might be produced by a single corrected myonucleus (3). AAV, adenoviral-associated virus; CRISPR, clustered regularly interspaced short palindromic repeats; DMD, Duchenne muscular dystrophy; NHEJ, non-homologous end joining.

It is important to realize that, through the typical process of cell fusion, each rare dystrophin-edited myoblast can contribute to the myofibers’ myonuclei, which would produce thousands of copies of dystrophin mRNA; if translated into protein, this truncated dystrophin will appear in the entire muscle fiber. Moreover, before fusion into myofibers, each rare dystrophin-edited myoblast is capable of proliferation and migration, thus having the capacity to contribute myonuclei to multiple myofibers in the treated tissue ( Figure 2). It is yet to be determined whether and to what degree CRISPR-Cas9 gene editing is effective in quiescent muscle stem cells (satellite cells) as opposed to the more differentiated proliferating progenitors (myoblasts), which typically exit the cell cycle to form myotubes and are unlikely to massively contribute to the stem cell pool that maintains and repairs muscle throughout the lifespan.

DMD and other X-linked genetic diseases do not have the second allele for natural HDR, hence there is no physiologic possibility to restore dystrophin (and X-linked mutations that cause genetic diseases in males, generally speaking) to the wild-type sequence. However, via the nanoparticle approach, the donor DNA template was successfully delivered in complex with the Cas9 and gRNA to the skeletal muscle of MDX mice, which produced *in vivo* HDR-based editing of mutant dystrophin to its wild-type sequence ^28^. Of note, in individual DMD patients, there are various mutations of many classes—missense, nonsense, small and large deletions, and so on—which might require patient-specific gRNA and donor DNA designs for the best treatment. At the same time, even truncated NHEJ-produced dystrophin that is expressed at partial levels might restore the viability of the muscle fibers, converting DMD to milder Becker’s muscular dystrophy where partial dystrophin protein is present in muscles of afflicted individuals ^29^. Improvement in cell viability by partial dystrophin is due to normalization of the membrane-associated dystrophin glycoprotein complex that normalizes Ca ^2+^ signaling and enhances the resilience of myofibers to contractile stress ^30– 33^. These observations underlie the strategy of using NHEJ to cut out the STOP codon in mutated dystrophin, targeting the 5′ to 3′ of the mutated sequences of dystrophin, and producing truncated exon-skipped, returned-to-frame genes. However, the *in vivo* method to treat DMD through exon skipping (without CRISPR-Cas9), which was approved by the FDA and was conducted by Sarepta (Exondys 51/eteplirsen) in 2016, resulted in only a marginal presence of dystrophin protein and could be applied only to patients with a specific mutation in the dystrophin gene ^34^. So the company switched to an older approach: gene therapy through delivery of dystrophin mini-gene cDNA, which recently yielded much better results ^35^. Of note, in the search for a broad gene editing therapy that can be applied to diverse DMD-causing mutations, a novel CRISPR-Cas9 NHEJ strategy was recently put forward ^36^.

Summarily, DMD is a recessive disease, in which any induction of dystrophin protein expression might be significant, particularly if it is above the percentage of revertant fibers, which are infrequently generated by rare cells with spontaneous somatic mutation(s) that result in dystrophin protein re-expression ^37^.

Although this review is focused on genetic myopathies, there are, of course, other genetic diseases in which, as in DMD, some disease-modifying activity might be expected, even with 1% to 2% of actual genomic DNA gene correction. For example, in autosomal recessive alpha 1 antitrypsin deficiency (A1AT), the Glu342Lys causative point mutation is in the SERPINA1 gene that is located on chromosome 14q and encodes liver-secreted serine protease inhibitor ^38, 39^. Any increase in the protease inhibitor might be helpful for attenuating the disease; moreover, the wild-type allele of SERPINA1 is available for HDR and physiologic correction of the mutation to the wild-type sequence. On the other side of this coin, there is a possibility of inadvertently altering the wild-type non-affected allele, which should be avoided at all costs; for example, the gRNA design becomes vitally stringent. Another detail of A1AT and liver diseases in general is that hepatocytes are polyploid, introducing the potential for interaction between edited and non-edited genomic DNA in sister chromatids and between identical chromosomes, complicating the gene editing/altering outcomes. PiZ transgenic mice that model this disease have high levels of human mutated SERPINA1 protein, and these animals manifest liver fibrosis and hepatocellular carcinoma ^40^. Very promising pre-clinical data were recently obtained by using two AAVs to deliver CRISPR-Cas9 components to mice that model A1AT *in vivo*: one with Cas9 and the second with a gRNA and donor DNA ^41^.

In contrast to DMD, A1AT, and so on, genetic disease in which even minimal *in vivo* effectiveness in gene correction might produce gradual cumulative attenuation of pathology, there are dominant negative diseases, exemplified by the myotonic dystrophies, in which meaningful intervention would require gene editing in a large volume of tissue.

Myotonic dystrophies (DM1 and DM2) are autosomal dominant neuromuscular genetic diseases ^42^. These disorders are systemic, they cause skeletal and heart muscle problems, and in DM1 but not DM2 pathology there are also brain problems and congenital manifestation.

Molecularly, in DM1, there is expansion of several hundreds or even thousands of a (CTG•CAG)n repeat sequence in the 3′ untranslated region of the myotonic dystrophy protein kinase ( *DMPK*) gene; in DM2, long expansions occur in a (CCTG•CAGG)n repeat sequence in the first intron of the gene for cellular nucleic acid binding protein (CNBP) ^43^. Resulting intranuclear accumulation of toxic RNA transcripts with these repetitive elements causes abnormal binding and affects the expression level and intracellular distribution of RNA binding proteins, notably of MBNL1 (which becomes diminished) and CUGBP1 (which becomes increased). Changes in the levels of CUGBP1 and MBNL1 percolate through altered RNA processing (RNA splicing, polyadenylation, and nucleocytoplasmic transport) of other gene products, which is regulated by these proteins ^42, 43^.

On one hand, CRISPR-Cas9-based approaches for treating myotonic dystrophies are facilitated, since NHEJ is applicable to promote the cleavage to sites 5′ and 3′ of the repeat. This is relatively easier than the HDR approach. But, on the other hand, the disease-causing repeats and resulting toxic RNAs are dominant negative inhibitors of cell function and are present in large tissues; thus, removing only a small percentage of them might not be sufficient for disease-modifying activity. Because DM1 and DM2 are mostly late-onset, genome editing of muscle stem cells (or even a small fraction of those cells) might help to halt disease progression and contribute to improvement in the long term. The numbers of true self-renewing stem cells and the regenerative capacity of such cells generally decline in skeletal muscle and other tissues with a person’s age, pointing toward the idea to target DMD, myotonic dystrophies, and genetic diseases in general as early as is practical.

Interestingly, even in myotonic dystrophy DM1, in which seemingly any reduction in the number of excessive repeats is therapeutic, only the removal of the entire expanded DNA sequence through precise CRISPR-Cas9 cuts on both sides was productive, whereas single cuts and less controlled NHEJ only increased genomic instability and pathology ^44^. Such findings point toward the idea that CRISPR-Cas9 machinery that contains multiple gRNAs and, in some cases, donor DNA template in addition to Cas9 might be needed for meaningful therapies as compared with the simpler single-target DNA DSB producing CRISPR-Cas9.

## Future directions

Moving forward, the field of CRISPR-Cas9 editing is working in a number of scientific directions. In particular, there is great interest in developing non-viral delivery strategies for delivering the CRISPR-Cas9 components into cells and *in vivo*. Delivery vehicles composed of solid lipid nanoparticles ^45^, lipofectamine ^46^, gold nanoparticles ^47^, and cationic polymers ^48^ have all been investigated for non-viral delivery of Cas9 and gRNA and have been able to efficiently inactivate genes *in vivo* in a variety of tissues. In addition, gold nanoparticles complexed to Cas9, gRNA(s), and dDNA (termed CRISPR-Cas9-Gold) have been developed and were able to generate HDR *in vivo*, in the muscle tissue, with an HDR efficiency of 5% ^28^. CRISPR-Cas9-Gold was also able to mitigate immune recognition (no AAV) and had lower off-target effects (no continuous Cas9 gene expression) as compared with the viral vectors ^28^.

Nanoparticles enable repetitive cumulative treatments, so even with a small percentage of gene editing in each, the overall positive effects would increase with the number of administrations (without expanding the DNA DSBs and immune reactions). The Cas9RNP that is delivered by nanoparticle will, of course, be degraded in the cell, but published work shows that effective gene editing takes place through this approach using the limited half-life of Cas9RNP ^28^. And while continuous expression of Cas9 cDNA and gRNA logically increases the change of gene editing in a given cell, there is a concomitant increase in genomic instability over time. Another positive of CRISPR-Cas9 nanoparticles is that there is no component’s size limitation in contrast to such limitation in cloning CRISPR-Cas9 into viral vectors. Though promising, nanoparticle delivery vehicles for CRISPR-Cas9 need to be optimized with respect to biodegradability and tissue targeting-homing from the bloodstream to tissues in need of repair.

As already mentioned, it would be important to target CRISPR-Cas9 to tissue stem and progenitor cells: the regenerative subset that is capable of effective DNA repair and thus CRISPR-Cas9-based gene editing. Importantly, because stem cells self-renew and differentiate, each gene correction would not only produce healthy functional tissue but also propagate in time and space through stem cell self-renewal and proliferation of tissue-resident progenitor cells. Finally, more knowledge is needed on the abilities of specific cell types and of cells at different stages of their lineage progression to function as targets for CRISPR-Cas9 editing and to mediate dsDNA break repair ^49, 50^.

Taking into account the limitations of NHEJ and the abilities of HDR to correct genes to their wild-type sequences by CRISPR-Cas9, several approaches are being developed to inhibit NHEJ and promote HDR ^51– 53^. To increase the effectiveness of HDR, which typically is a slow, inefficient process, ectopic expression of Rad52, linear DNA templates, and small-molecule approaches were successfully tried ^52, 54, 55^.

In attempts to minimize the off-target effects of CRISPR-Cas9 and limit the undesired genome damage, deliberate inhibitors of Cas9 have been developed ^56^, and computational platforms for predicting the on-target versus off-target sites for CRISPR-Cas9 gRNAs have been proposed ^54, 57, 58^.

Finally, the progress of CRISPR-Cas9 toward the clinic now relies heavily on two-dimensional cell cultures and rodent models (both significantly different from human tissues). More advances are needed in testing and optimizing CRISPR-Cas9 for both NHEJ and HDR in human cells and tissues, and organ chips present a great opportunity in this regard. Namely, the recent development of three-dimensional human micro-tissues ^59– 63^ allows one to examine CRISPR-Cas9 applications in disease-relevant cells and also when different cell types are present in an environment that emulated the afflicted organ, at least with respect to the “vasculature” and possibly the “innervation”. In a similar vein, the work with mouse models of human genetic diseases is to progress toward large animals; on this path, work in a canine DMD model that is phenotypically more similar to human disease than the MDX mice was successful in 2% to 3% of dystrophin editing by CRISPR-Cas9 (as determined by genomic DNA sequencing). Such genome editing in two studied animals produced re-expression of dystrophin protein throughout the body musculature after infusions of AAV-Cas9 and AAV-gRNA cassettes into immunocompromised 1-month-old pups with their growing muscles and hence proliferative myogenic cells ^64^.

Summarily, the field of CRISPR-Cas9 is blossoming, and this scientific method has immense potential to become therapeutic; to realize this potential, CRISPR-Cas9 has to be improved with respect to delivery to regenerative dividing cells, enabled tissue targeting, enhanced HDR or other forms of precision gene editing, and reduction-elimination of the CRSIPR-caused genomic damage.
